# Novel 2-Phenoxyanilide Congeners Derived from a Hit Structure of the TCAMS: Synthesis and Evaluation of Their *in Vitro* Activity against *Plasmodium falciparum*

**DOI:** 10.3390/molecules21020223

**Published:** 2016-02-17

**Authors:** Thomas Weidner, Abed Nasereddin, Lutz Preu, Johann Grünefeld, Ron Dzikowski, Conrad Kunick

**Affiliations:** 1 Institut für Medizinische und Pharmazeutische Chemie, Technische Universität Braunschweig, Beethovenstraße 55, 38106 Braunschweig, Germany; t.weidner@tu-braunschweig.de (T.W.); l.preu@tu-braunschweig.de (L.P.); j.gruenefeld@tu-braunschweig.de (J.G.); 2 Department of Microbiology and Molecular Genetics, IMRIC, The Kuvin Center for the Study of Infectious and Tropical Diseases, The Hebrew University-Hadassah Medical School, Jerusalem 91120, Israel; abedn@ekmd.huji.ac.il (A.N.); rond@ekmd.huji.ac.il (R.D.); 3 Center of Pharmaceutical Engineering (PVZ), Technische Universität Braunschweig, Franz-Liszt-Straße 35A, 38106 Braunschweig, Germany

**Keywords:** diarylether, luciferase, Malaria, 2-phenoxyanilide, *Plasmodium falciparum*, TCAMS, TCMDC-137332

## Abstract

The Tres Cantos Antimalarial Compound Set (TCAMS) is a publicly available compound library which contains 13533 hit structures with confirmed activity against *Plasmodium falciparum*, the infective agent responsible for malaria tropica. The TCAMS provides a variety of starting points for the investigation of new antiplasmodial drug leads. One of the promising compounds is TCMDC-137332, which seemed to be a good starting point due to its antiplasmodial potency and its predicted physicochemical properties. Several new analogues based on a 2-phenoxyanilide scaffold were synthesized by standard amide coupling reactions and were fully characterized regarding their identity and purity by spectroscopic and chromatographic methods. Furthermore, the results of the biological evaluation of all congeners against *Plasmodium falciparum* NF54 strains are presented. The findings of our *in vitro* screening could not confirm the presumed nanomolar antiplasmodial activity of TCMDC-137332 and its derivatives.

## 1. Introduction

Malaria is still one of the most severe infectious diseases. Approximately 3.2 billion people are at risk of being infected. Despite a decreasing number of mortal cases in the last decade due to better vector control and artemisinine-based combination therapy (ACT), still 584,000 deaths caused by malaria infection were reported in 2013 [1]. Although there are effective chemotherapeutics for the treatment of malaria, the discovery of new drugs is important due to the increasing resistances against available drugs [2,3]. A good strategy to circumvent the problem of resistance is to develop compounds acting with new mechanisms of action. Hence there is an urgent need for compounds with novel chemotypes which differ from scaffolds of existing drugs.

Major sources for drug discovery are phenotypic screening libraries, providing a huge number of new starting points. In the last few years extensive libraries with compounds showing antimalarial activity have been published by Novartis [4], St. Jude’s Children Research Hospital [5], the Medical Research Council Technology [6] and GlaxoSmithKline (GSK) [7]. The latter was derived from a high throughput screening (HTS) of nearly 2 million substances of the GSK corporate collection. 13,533 of these, known as the Tres Cantos Antimalarial Compound Set (TCAMS), were active against the malaria parasite *Plasmodium falciparum* and inhibited the parasite growth by at least 80% at 2 µM concentration. *In silico* clustering and filtering of the TCAMS set performed by Calderon *et al.* resulted in 552 compounds which were declared as “quality starting points from the TCAMS” [8]. Since then, a few of the compound classes have been further examined, e.g., cyclopropylcarboxamides [9], 2-amino-1-phenylethanols [10], aminohydantoines [11] or carbamoyltriazoles [12].

**Figure 1:**
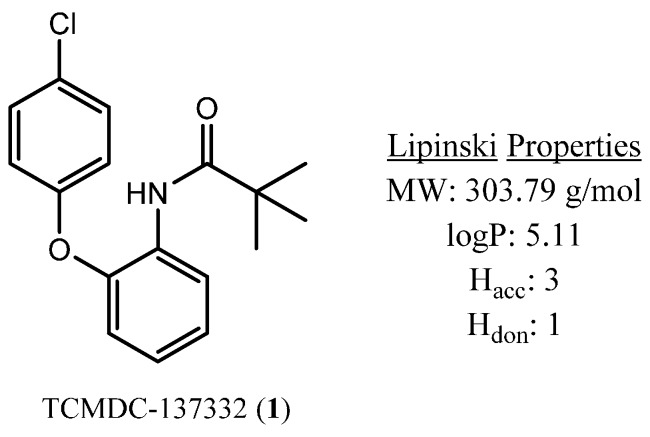
Molecular structure of TCMDC-137332 and its properties concerning Lipinski’s rules for orally available drugs; chemicalize.org was used for calculation of logP [15].

One of the 552 promising compounds is TCMDC-137332 (**1**), which has an estimated IC_50_ value of 7 nM and therefore seemed to be one of the most potent structures of this collection [7,8]. We decided to further investigate this compound which matches nearly all criteria of the Lipinski rules for orally available drugs [13] (Figure 1) and is structurally dissimilar to currently known antimalarials [14].

## 2. Results and Discussion

### 2.1. Chemistry

**Figure 2:**
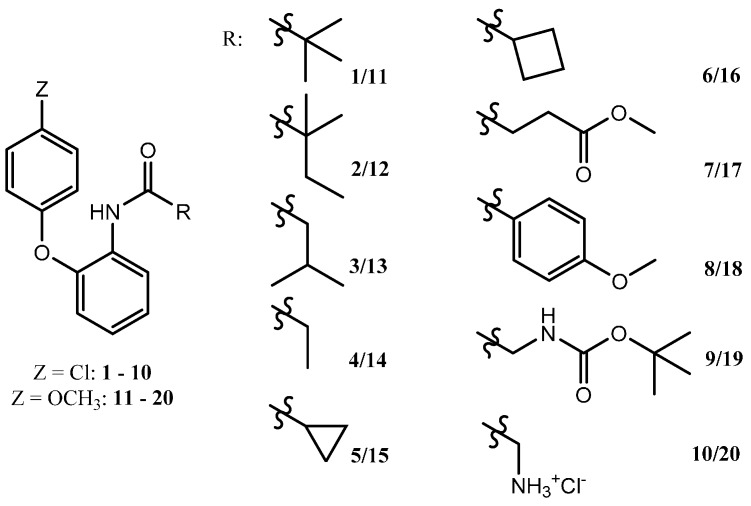
Structures of TCMDC-137332 (**1**) and its congeners (**2**–**19**).

To proof its activity, we resynthesized TCMDC-137332. We also prepared a series of congeners, all having a 2-phenoxyanilide scaffold (Figure 2). The first group of ten compounds was substituted with chlorine in para position of the phenoxy residue, like in the scaffold of TCMDC-137332 (**1**–**10**). Another equivalent series was synthesized, in which the chlorine was replaced by a more polar methoxy group (**11**–**20**).

**Scheme 1:**
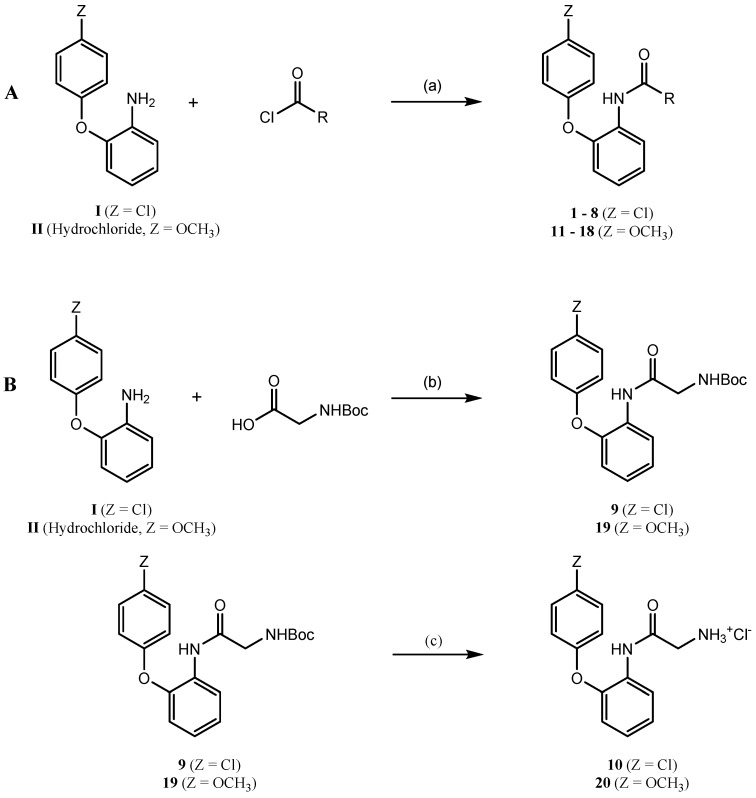
(**A**) Synthesis of 2-phenoxyanilides **1**–**8** and **11**–**18**. *Reaction conditions*: (a) triethylamine, toluene, 0 °C → rt. Residues R: see Figure 2; (**B**) Synthesis of 2-phenoxyanilides **9**, **10**, **19** and **20**. *Reaction conditions*: (b) PyBOB, DIPEA, dichloromethane, 0 °C → rt; (c) 1. Trifluoroacetic acid, dichloromethane, rt, 2. Hydrochloric acid (37%)/propan-2-ol (1:1).

The synthesis of phenoxyanilides **1**–**8** and **11**–**18** was achieved by standard amide coupling reactions of commercially available 2-(4-chlorophenoxy)aniline **I** or 2-(4-methoxyphenoxy)aniline hydrochloride **II** with acid chlorides in presence of triethylamine, as outlined in Scheme 1A. Compounds **9** and **19** were synthesized by the reaction of 2-(4-substituted)phenoxyanilines and Boc-protected glycine in the presence of PyBOP (benzotriazol-1-yl-oxytripyrrolidinophosphonium hexafluorophosphate) together with DIPEA (Diisopropylethylamine). Cleavage of the protecting group was accomplished by treatment of **9** and **19**, respectively, with trifluoroacetic acid and subsequent precipitation of the hydrochloride salts **10** and **20** with a hydrochloric acid solution in propan-2-ol (Scheme 1B). The compounds were synthesized in satisfactory to excellent yields (53%–93%) and the purity (determined with elemental analyses and HPLC) of most of the products was sufficient for biological evaluation even before the final purification step (recrystallization, flash chromatography).

### 2.2. Calculations of Rule-of-Five-properties

We determined the properties concerning Lipinski’s Rule of Five (RO5) for orally active drugs for all derivatives. The RO5 can be a useful reference to predict the quality of a structure to be an orally available drug-like compound [13,16], which was outlined by Calderon *et al.* for TCMDC-137332 [8]. Considering the molecular weight (<380 g/mol), the amount of H-bond acceptors (≤6) and H-bond donors (≤3), the values of every compound in this series comply with the proposed values of Lipinski *et al.* [13]. Estimation of lipophilicity has been carried out using chemicalize.org for calculation of logP [15]. According to the RO5 the logP should have a maximum value of 5. The calculated logP compared to TCMDC-137332 was reduced by replacement of the *tert*-butyl residue by smaller residues, cyclic aliphatic residues like cyclopropyl- or cyclobutyl-groups, and more polar groups. Introducing a methoxy group instead of the chlorine residue also reduced the calculated logP value. Some examples of the calculated octanol/water partition coefficients are outlined in Table 1.

### 2.3. In Vitro Assay

**Figure 3:**
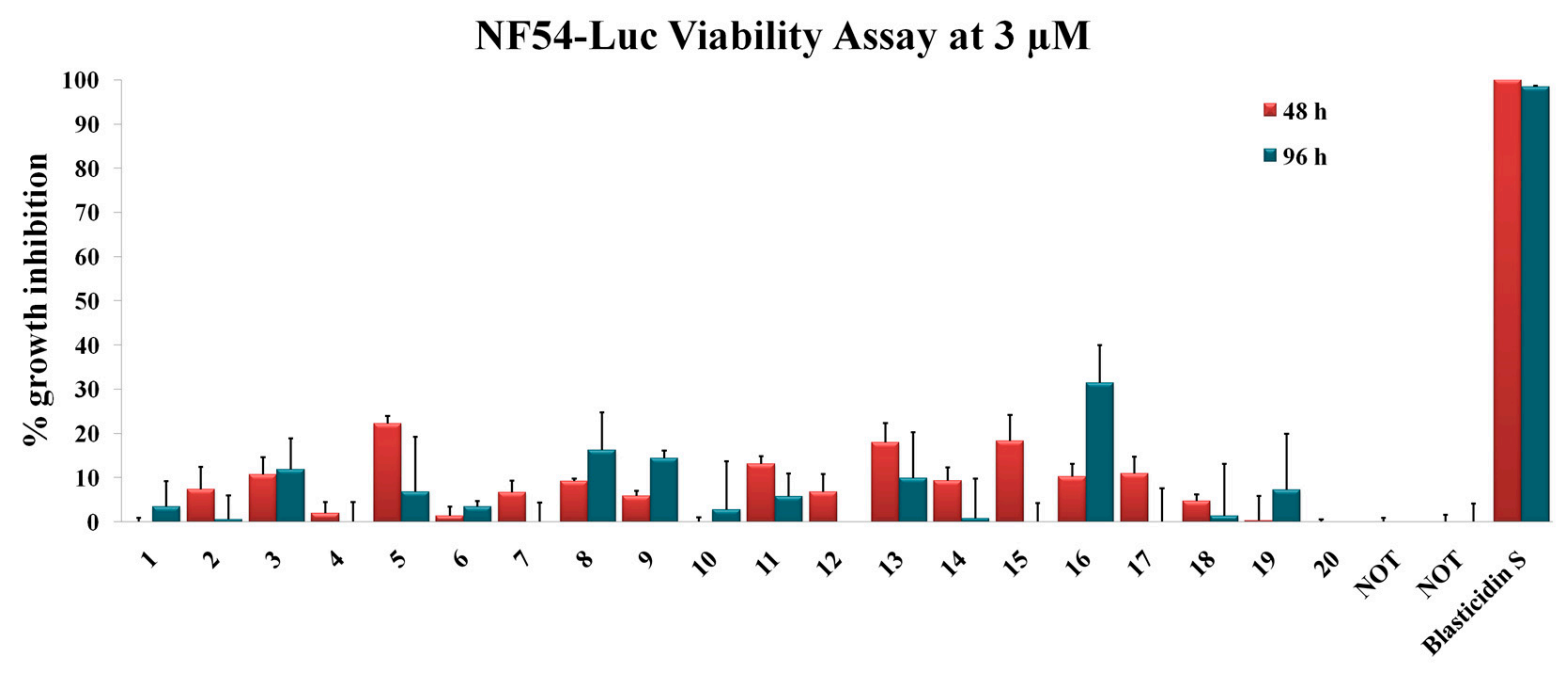
Results of the *in vitro* assay of derivatives **1**–**20** in 3 µM concentration against *P. falciparum* NF54-Luc parasite line with 48 h (**red**) and 96 h (**blue**) incubation time. NOT: no treatment; Blasticidin S: antibiotic used as antiplasmodial standard.

After the full spectroscopic characterization of all congeners and validation of their purity by elemental analyses and chromatographic methods *in vitro* assays were carried out using *P. falciparum* NF54-*luc* erythrocyte stage parasites expressing luciferase. The derivatives **1**–**20** were examined in 3 µM concentration performing viability assays measuring the bioluminescence in a luciferase assay system as described earlier [17,18,19,20]. A first screening, in which the cultures were incubated for 48 h, gave surprisingly low inhibition rates of the parasite growth (see Figure 3). With 22% reduction of the parasite viability, **5** was the most effective compound in this screening, followed by **13** and **15** with about 18% inhibition. The resynthesized TCMDC-137332 (**1**) failed to show any antiplasmodial activity, just as compounds **10** and **20** which contain polar aminomethyl residues. Due to the unexpected outcome of the first screening session, we performed another assay in which the cultures were incubated for a longer period (96 h), to identify slow acting compounds. However, the prolongation of the incubation time did not improve the inhibitory effect of the derivatives on the parasite growth significantly, the only exception being compound **16**, which diminished the proliferation of *P. falciparum* NF54 by 31%. Compared to the first screening, **16** showed a 3-fold better inhibition rate, indicating that it is probably a rather slow acting inhibitor. However, the results of most of the derivatives were slightly inferior compared to the first screening. The assumed high antiplasmodial activity of TCMDC-137332 (**1**) could not be confirmed by these assays.

## 3. Experimental Section

### 3.1. Apparatus and Materials

Starting materials were purchased from the suppliers indicated below and were used without further purification. Pivaloyl chloride was from Merck-Schuchardt (Hohenbrunn, Germany), 2,2-dimethylbutyryl chloride, 3-methylbutyryl chloride and 4-methoxybenzoyl chloride were from Alfa-Aesar (Karlsruhe, Germany), propyl chloride, cyclopropanecarbonyl chloride, cyclobutanecarbonyl chloride and methyl succinyl chloride were from Acros (Geel, Belgium), Boc-glycine was from Aldrich (Steinheim, Germany), 2-(4-chlorophenoxy)aniline was from Enamine (Monmouth Jct., NJ, USA), 2-(4-methoxyphenoxy)aniline hydrochloride was from Fluorochem (Hadfield, UK). Toluene and dichloromethane were dried by published methods before usage [21]. Melting points were determined on an electric variable heater (Electrothermal IA9200, Bibby Scientific, Stone, UK) in open glass capillaries and are uncorrected. IR spectra were recorded as KBr disks on a Thermo Nicolet FT-IR 200 (Thermo Nicolet, Madison, WI, USA). ^1^H-NMR spectra and ^13^C-NMR spectra were recorded on BrukerAvance DRX-400 and BrukerAvance II-600 instruments (Bruker Corporation, Billerica, MA, USA) (at the NMR laboratories of the Chemical Institutes of the Technische Universität Braunschweig). Chemical shifts were recorded as δ values in ppm and are referenced to an internal standard tetramethylsilane. Signals in ^13^C spectra were assigned based on the result of ^13^C DEPT135 experiments. Elemental analyses were determined on a CE Instruments FlashEA 1112 elemental analyzer (Thermo Quest, San Jose, CA, USA). Mass spectra were recorded on a Finnigan-MAT 95 (Thermo Finnigan MAT, Bremen, Germany). Accurate measurements were conducted according to the peak match method using perfluorokerosene (PFK) as an internal mass reference. (EI) MS: ionization energy 70 eV (Department of Mass Spectrometry of the Chemical Institutes of the Technische Universität Braunschweig).TLC: Polygram Sil G/UV_254_ (Macherey-Nagel, Düren, Germany), 40 mm × 80 mm, visualization by UV illumination (254 and 366 nm). Purity was determined by HPLC using isocratic and gradient elution performed on Merck Hitachi Elite LaChrom systems (Hitachi High Technologies Inc., San Jose, CA, USA): pump L-2130, autosampler L-2200, diode array detector L-2450 (isocratic elution) or UV detector L-2400 (gradient elution), organizer box L-2000; column, Merck LiChroCART 125-4, LiChrosphere 100, RP 18, 5 μm (Merck, Darmstadt, Germany); flow rate 1.000 mL/min; detection wavelength: 254 and 280 nm (isocratic elution) and 254 nm (gradient elution); AUC, % method; time of detection 15 min (isocratic elution) or 20 min (gradient elution), retention time (t_R_); dead time (t_M_) related to DMSO. For isocratic runs, mixtures of ACN and water or mixtures of ACN and buffer were used. For all gradient runs, mixtures of ACN and water were used (gradient: 0–2 min: 10% ACN, 2–12 min: 10% → 90% ACN, linear, 12–20 min 90% ACN). Preparation of H_2_O + (Et_3_NH)_2_SO_4_ buffer (pH 2.7) for isocratic HPLC: triethylamine (20.0 mL) and sodium hydroxide (242 mg) were dissolved in water to 1 L. The solution was adjusted to pH 2.7 by addition of sulfuric acid. All compounds which were biologically tested were of >95% purity. Absorption maxima (λ_max_) were extracted from the spectra recorded by the DAD in the HPLC peak maxima in isocratic runs (software, EZ Chrom Elite Client/server, version 3.1.3., Scientific Software Inc., Pleasanton, CA, USA).

### 3.2. Chemical Synthesis and Characterization of Compounds **1**–**20**

#### General Procedure for the Synthesis of Compounds **1**–**8**

To a stirred and cooled solution of 2-(4-chlorophenoxy)aniline (330 mg, 1.50 mmol) and triethylamine (230 µL, 1.65 mmol) in toluene (5 mL), the appropriate acid chloride (pivaloyl chloride, 2,2-dimethylbutyryl chloride, 3-methylbutyryl chloride, propyl chloride, cyclopropanecarbonyl chloride, cyclobutanecarbonyl chloride, methyl succinyl chloride, 4-methoxybenzoyl chloride) (1.65 mmol) was added. Subsequently the reaction mixture was allowed to warm to room temperature. The progress of the reaction was monitored by TLC. After 2–9 h the reaction mixture was extracted with a saturated sodium hydrogen carbonate solution, with a hydrogen chloride solution (10%), with brine and finally with water. Afterwards the organic solution was dried over sodium sulfate and evaporated under reduced pressure. The residue was further purified by recrystallization or column chromatography over silica gel.

*N-[2-(4-Chlorophenoxy)phenyl]-2,2-dimethylpropanamide* (**1**): Crystallization from ethanol (70%) yielded slightly brown needles (277 mg, 0.92 mmol, 61%); m.p.: 99–100 °C ; IR (KBr): $\bar{\nu }$ [cm^−1^] = 3336 (br, N-H), 1663 (s, C=O); ^1^H-NMR: (400 MHz, DMSO-*d*_6_) δ [ppm] = 1.05 (s, 9H, C(C*H*_3_)_3_), 6.80–7.01 (m, 2H, arom. H), 6.99–7.15 (m, 1H, arom. H), 7.15–7.32 (m, 2H, arom. H), 7.27–7.48 (m, 2H, arom. H), 7.56–7.78 (m, 1H, arom. H), 8.72 (s, 1H, N*H*); ^13^C-NMR: (101 MHz, DMSO-*d*_6_) δ [ppm] = 26.95 (3C, *C*H_3_), 118.40 (2C), 120.46, 124.63, 126.13, 126.62, 129.49 (2C) (*C*H), 38.12, 126.38, 130.28, 147.84, 155.91 (*C*), 176.13 (C=O); C_17_H_18_ClNO_2_ (303.78): calcd. C 67.21, H 5.97, N 4.61, found C 67.13, H 6.00, N 4.51; EI-MS: *m*/*z* (%): 303.1 [M]^+^ (28), 176.1 [M − 127]^+^ (100); HPLC: 99.7% at 254 nm, 99.9% at 280 nm; t_R_ = 7.42 min, t_M_(DMSO) = 1.06 min (ACN/H_2_O 60:40), λ_max_ [nm] = 230, 275; HPLC-gradient: 99.4%, t_R_ = 13.62 min, t_M_(DMSO) = 1.28 min.

*N-[2-(4-Chlorphenoxy)phenyl]-2,2-dimethylbutanamide* (**2**): Crystallization from methanol/water (33:10) yielded a beige solid (332 mg, 1.05 mmol, 70%); m.p.: 69–70 °C ; IR (KBr): $\bar{\nu }$ [cm^−1^] = 3316 (br, N-H), 1657 (s, C=O); ^1^H-NMR: (400 MHz, DMSO-*d*_6_) δ [ppm] = 0.67 (t, *J* = 7.4 Hz, 3H, C*H*_3_), 1.01 (s, 6H, C(C*H*_3_)_2_), 1.47 (q, *J* = 7.4 Hz, 2H, C*H*_2_), 6.87–6.96 (m, 2H, arom. H), 7.03–7.13 (m, 1H, arom. H), 7.17–7.27 (m, 2H, arom. H), 7.34–7.43 (m, 2H, arom. H), 7.59–7.68 (m, 1H, arom. H), 8.70 (s, 1H, N*H*); ^13^C-NMR: (101 MHz, DMSO-*d*_6_) δ [ppm] = 8.82, 24.54 (2C) (*C*H_3_), 32.82 (*C*H_2_) 118.49 (2C), 120.46, 124.63, 126.16, 126.74, 129.55 (2C) (*C*H), 42.42, 126.43, 130.29, 147.98, 155.99 (*C*), 175.48 (C=O); C_18_H_20_ClNO_2_ (317.81): calcd. C 68.03, H 6.34, N 4.41, found C 68.09, H 6.22, N 4.28; EI-MS: *m*/*z* (%): 317.1 [M]^+^ (28), 190.1 [M − 127]^+^ (100); HPLC: 98.7% at 254 nm, 99.0% at 280 nm; t_R_ = 9.82 min, t_M_(DMSO) = 1.06 min (ACN/H_2_O = 60:40), λ_max_[nm] = 232, 275; HPLC-Gradient: 97.3%, t_R_ = 14.17 min, t_M_(DMSO) = 1.28 min.

*N-[2-(4-Chlorophenoxy)phenyl]-3-methylbutanamide* (**3**): Purification by column chromatography (dichloromethane/methanol 200:1) yielded a slightly yellow solid (418 mg, 1.38 mmol, 92%); m.p.: 69–71 °C; IR (KBr): $\bar{\nu }$ [cm^−1^] = 3291 (m, N-H), 1655 (s, C=O); ^1^H-NMR: (400 MHz, DMSO-*d*_6_): δ [ppm] = 0.81 (d, *J* = 6.7 Hz, 6H, C*H*_3_), 1.92 (hept, *J* = 6.8 Hz, 1H, C*H*(CH_3_)_2_), 2.14 (d, *J* = 7.2 Hz, 2H, C*H*_2_), 6.79–7.08 (m, 3H, arom. H), 7.08–7.25 (m, 2H, arom. H), 7.28–7.51 (m, 2H, arom. H), 7.88 (dd, *J* = 7.6 Hz, 2.2 Hz, 1H, arom. H), 9.38 (s, 1H, N*H*); ^13^C-NMR (101 MHz, DMSO-*d*_6_) δ [ppm] = 22.10 (2C, *C*H_3_), 44.93 (*C*H_2_), 25.60, 119.28 (2C), 119.83, 124.34, 124.87, 125.25, 129.56 (2C) (*C*H), 126.67, 130.18, 147.04, 155.98 (*C*), 170.89 (*C=*O); C_17_H_18_ClNO_2_ (303.78): calcd. C 67.21, H 5.97, N 4.61, found C 66.95, H 6.02, N 4.39; EI-MS: *m*/*z* (%): 303.1 [M]^+^ (16), 219.0 [M − 84]^+^ (100); HPLC: 98.1% at 254 nm, 99.0% at 280 nm; t_R_ = 6.03 min, t_M_(DMSO) = 1.06 min (ACN/H_2_O = 60:40), λ_max_ [nm] = 249; HPLC-gradient: 95.6%, t_R_ = 13.23 min, t_M_(DMSO) = 1.28 min.

*N-[2-(4-Chlorophenoxy)phenyl]propanamide* (**4**): Purification by column chromatography (petroleum ether/ethyl acetate 4:1) yielded a colorless solid (265 mg, 0.96 mmol, 63%); m.p.: 99–100 °C; IR (KBr): $\bar{\nu }$ [cm^−1^] = 3330 (br, N-H), 1675 (s, C=O); ^1^H-NMR: (400 MHz, DMSO-*d*_6_) δ [ppm] = 0.98 (t, *J* = 7.6 Hz, 3H, C*H*_3_), 2.29 (q, *J* = 7.6 Hz, 2H, C*H*_2_), 6.93–7.02 (m, 3H, arom. H), 7.08–7.22 (m, 2H, arom. H), 7.37–7.46 (m, 2H, arom. H), 7.91–7.98 (m, 1H, arom. H), 9.39 (s, 1H, N*H*); ^13^C-NMR: (101 MHz, DMSO-*d*_6_): δ [ppm] = 9.65 (*C*H_3_), 29.03 (*C*H_2_), 119.53 (2C), 124.24, 124.43, 125.02, 129.59 (2C) (*C*H), 126.78, 130.19, 146.93, 155.85 (*C*), 172.31 (C=O), one signal missing in ^13^C-NMR; C_15_H_14_ClNO_2_ (275.73): calcd. C 65.34, H 5.12, N 5.08, found C 65.33, N 4.98, H 5.02; EI-MS: *m*/*z* (%): 275.1 [M]^+^ (18), 219.0 [M − 56]^+^ (100); HPLC: 98.9% at 254 nm, 99.4% at 280 nm; t_R_ = 3.95 min, t_M_(DMSO) = 1.06 min (ACN/H_2_O = 60:40), λ_max_ [nm] = 248, 274; HPLC-gradient: 96.0%, t_R_ = 12.23 min, t_M_(DMSO) = 1.28 min.

*N-[2-(4-Chlorophenoxy)phenyl]cyclopropanecarboxyamide* (**5**): Crystallization from ethanol (70%) yielded beige needles (387 mg, 1.34 mmol, 90%); m.p.: 100–102 °C; IR (KBr): $\bar{\nu }$ [cm^−1^] = 3371, 3332 (m, N-H), 1663 (s, C=O); ^1^H-NMR: (400 MHz, DMSO-*d*_6_): δ [ppm] = 0.67–0.77 (m, 4H, C*H_2_*), 1.95 (td, *J* = 7.5 Hz, 3.9 Hz, 1H, C*H*), 6.94 (dd, *J* = 7.5 Hz, 2.1 Hz, 1H, arom. H), 6.96–7.03 (m, 2H, arom. H), 7.06–7.26 (m, 2H, arom. H), 7.37–7.41 (m, 2H, arom. H), 7.96 (dd, *J* = 7.7 Hz, *J* = 2.1 Hz, 1H, arom. H), 9.71 (s, 1H, N*H*); ^13^C NMR (101 MHz, DMSO-*d*_6_) δ [ppm] = 7.16 (2C, *C*H_2_), 13.86, 119.14, 119.70 (2C), 124.01, 124.73, 129.55 (2C) (*C*H), 126.82, 130.09, 146.66, 155.71 (*C*), 171.99 (*C=*O); C_16_H_14_ClNO_2_ (287.74): calcd. C 66.79, H 4.90, N 4.87, found C 66.96, H 4.92, N 4.77; EI-MS: *m*/*z* (%): 287.1 [M]^+^ (21), 219.0 [M − 68]^+^ (100); HPLC: 98.4% at 254 nm, 98.3% at 280 nm; t_R_ = 4.40 min, t_M_(DMSO) = 1.06 min (ACN/H_2_O = 60:40), λ_max_ [nm] = 241; HPLC-gradient: 97.5%, t_R_ = 12.57 min, t_M_(DMSO) = 1.28 min.

*N-[2-(4-Chlorophenoxy)phenyl]cyclobutanecarboxyamide* (**6**): Purification by column chromatography (toluene/petroleum ether 10:1 → 20:1) yielded a slightly brown solid (246, 0.82 mmol, 53%): m.p.: 88–89 °C; IR (KBr): $\bar{\nu }$ [cm^−1^] = 3433 (br, N-H), 1657 (s, C=O); ^1^H-NMR: (400 MHz, DMSO-*d*_6_) δ [ppm] = 1.66–2.14 (m, 6H, C*H*_2_), 3.19–3.33 (m, 1H, C*H*), 6.90–7.03 (m, 3H, arom. H), 7.12–7.20 (m, 2H, arom. H), 7.36–7.45 (m, 2H, arom. H), 7.91 (dd, *J* = 7.4 Hz, 2.1 Hz, 1H, arom. H), 9.22 (s, 1H, N*H*); ^13^C-NMR: (101 MHz, DMSO-*d*_6_) δ [ppm] = 17.65, 24.47 (2C) (*C*H_2_), 38.88, 119.28 (2C), 119.78, 124.36, 124.74, 125.18, 129.55 (2C) (*C*H), 126.68, 130.18, 147.00, 155.87 (*C*), 173.09 (*C*=O); C_17_H_16_ClNO_2_ (301.77): calcd. C 67.66, H 5.34, N 4.64, found C 67.81, H 5.43, N 4.55; EI-MS: *m*/*z* (%): 301.1 [M]^+^ (20), 219.0 [M − 82]^+^ (100); HPLC: 99.4% at 254 nm, 99.8% at 280 nm; t_R_ = 5.86 min; t_M_(DMSO) = 1.06 min (ACN/H_2_O = 60:40), λ_max_ [nm] = 229; HPLC-gradient: 98.4%, t_R_ = 13.15 min; t_M_(DMSO) = 1.28 min.

*Methyl 4-{[2-(4-chlorophenoxy)phenyl]amino}-4-oxobutanoate* (**7**): Crystallization from methanol yielded a beige solid (278 mg, 0.83 mmol, 56%); m.p.: 109–110 °C; IR (KBr): $\bar{\nu }$ [cm^−1^] = 3341 (m, N-H),1721 (s, C=O, ester) 1687 (s, C=O, amide); ^1^H-NMR: (400 MHz, CDCl_3_): δ [ppm] = 2.35–2.97 (m, 4H, C*H_2_*), 3.67 (s, 3H, OC*H_3_*), 6.83 (dd, *J* = 8.1 Hz, 1.4 Hz, 1H, arom. H), 6.93–7.05 (m, 3H, arom. H), 7.12 (td, *J* = 7.8 Hz, 1.5 Hz, 1H, arom. H), 7.28–7.38 (m, 2H, arom. H), 7.91 (s, 1H, N*H*), 8.41 (d, *J* = 7.8 Hz, 1H, arom. H); ^13^C-NMR (101 MHz, CDCl_3_) δ [ppm] = 51.93 (*C*H_3_) 29.15, 32.27 (*C*H_2_), 117.77, 119.84 (2C), 121.14, 124.10, 124.44, 129.95 (2C) (*C*H), 128.98, 129.78, 145.22, 155.13 (*C*), 169.69 (*C=*O), 173.23 (*C=*O); C_17_H_16_ClNO_4_ (333.77): calcd. C 61.18, H 4.83, N 4.20, found: C 61.15, H 4.83, N 4.21; EI-MS: *m*/*z* (%): 333.1 [M]^+^ (12), 174.1 [M − 159]^+^ (100); HPLC: 99.3% at 254 nm, 99.7% at 280 nm; t_R_ = 3.44 min, t_M_(DMSO) = 1.06 min (ACN/H_2_O = 60:40), λ_max_ [nm] = 228; HPLC-gradient: 98.5%, t_R_ = 11.99 min, t_M_(DMSO) = 1.28 min.

*N-(2-(4-Chlorophenoxy)phenyl)-4-methoxybenzamide* (**8**): Crystallization from methanol yielded colorless needles (346mg,0.98 mol, 65%); m.p.: 101–102 °C; IR (KBr): $\bar{\nu }$ [cm^−1^] = 3420 (m, N-H), 1660 (s, C=O); ^1^H-NMR: (400 MHz, DMSO-*d*_6_): δ [ppm] = 3.81 (s, 3H, OC*H*_3_), 6.89–7.13 (m, 5H, arom. H), 7.17–7.30 (m, 2H, arom. H), 7.30–7.48 (m, 2H, arom. H), 7.65–7.76 (m, 1H, arom. H), 7.75–7.99 (m, 2H, arom. H), 9.69 (s, 1H, N*H*); ^13^C-NMR (101 MHz, DMSO-*d*_6_) δ [ppm] = 55.29 (*C*H_3_), 113.44 (2C), 119.36 (2C), 119.69, 124.15, 126.32, 126.83, 129.38 (2C), 129.48 (2C) (*C*H), 126.25, 126.64, 129.79, 149.02, 155.71, 161.80 (*C*), 164.69 (*C*=O); C_20_H_16_ClNO_3_ (353.80): calcd. C 67.90, H 4.56, N 3.96, found: C 67.78, H 4.43, N 3.95; EI-MS: *m*/*z* (%): 353.1 [M]^+^ (8), 135.0 [M − 218]^+^ (100); HPLC: 99.7% at 254 nm, 99.8% at 280 nm; t_R_ = 6.37 min, t_M_(DMSO) = 1.06 min (ACN/H_2_O = 60:40), λ_max_ [nm] = 266; HPLC-gradient: 99.0%, t_R_ = 13.37 min, t_M_(DMSO) = 1.28 min.

#### General Procedure for the Synthesis of Compounds **11**–**18**

To a stirred and cooled solution of 2-(4-methoxyphenoxy)aniline hydrochloride (1.50 mmol) and triethylamine (4.00 mmol) in toluene (5–8 mL), the appropriate acid chloride (pivaloyl chloride, 2,2-dimethylbutyryl chloride, 3-methylbutyryl chloride, propyl chloride, cyclopropanecarbonyl chloride, cyclobutanecarbonyl chloride, methyl succinyl chloride, 4-methoxybenzoyl chloride) (1.65 mmol) was added. Thereafter the reaction mixture was allowed to warm to room temperature. The progress of the reaction was monitored by TLC. After 4–24 h an aqueous work-up was performed similar to that of compounds **1**–**8**. The resulting residue was further purified by recrystallization or column chromatography over silica gel.

*N-[2-(4-Methoxyphenoxy)phenyl]-2,2-dimethylpropanamide* (**11**): Crystallization from ethanol (70%) yielded a colorless solid (401 mg, 1.34 mmol, 89%); m.p.: 70–71 °C; IR (KBr): $\bar{\nu }$ [cm^−1^] = 3317 (m, N-H), 1661 (s, C=O); ^1^H-NMR: (400 MHz, DMSO-*d*_6_): δ [ppm] = 1.11 (s, 9H, C(C*H_3_*)*_3_*), 3.72 (s, 3H, OC*H_3_*), 6.85–6.90 (m, 1H, arom. H), 6.92 (s, 4H, arom. H), 7.06–7.16 (m, 2H, arom. H), 7.69–7.78 (m, 1H, arom. H), 8.62 (s, 1H, N*H*); ^13^C-NMR (101 MHz, DMSO-*d*_6_) δ [ppm] = 27.06 (3C), 55.41 (C*H*_3_), 114.86 (2C), 118.35, 119.09 (2C), 123.25, 125.06, 125.40 (*C*H), 38.90, 129.48, 148.96, 149.97, 155.27 (*C*), 176.10 (*C=*O); C_18_H_21_NO_3_ (299.37): calcd. C 72.22, H 7.07, N 4.68, found C 72.21, H 7.25, N 4.73; EI-MS: *m*/*z* (%): 299.1 [M]^+^ (48), 176.1 [M − 123]^+^(100); HPLC: 99.4% at 254 nm, 99.4% at 280 nm; t_R_ = 5.21 min, t_M_(DMSO) = 1.06 min (ACN/H_2_O = 60:40), λ_max_ [nm] = 248; HPLC-Gradient: 99.3%, t_R_ = 12.88 min, t_M_(DMSO) = 1.28 min.

*N-[2-(4-Methoxyphenoxy)phenyl]-2,2-dimethylbutanamide* (**12**): Crystallization from ethanol (70%) yielded a slightly yellow solid (415 mg, 1.32 mmol, 88%): m.p.: 71–72 °C; IR (KBr): $\bar{\nu }$ [cm^−1^] = 3433 (m, N-H), 1674 (s, C=O); ^1^H-NMR: (400 MHz, DMSO-*d*_6_): δ [ppm] = 0.72 (t, *J* = 7.4 Hz, 3H, C*H_3_*), 1.07 (s, 6H, C(C*H*_3_)_2_), 1.51 (q, *J* = 7.4 Hz, 2H, C*H*_2_), 3.72 (s, 3H, OC*H*_3_), 6.83–6.92 (m, 1H, arom. H), 6.92 (s, 4H, arom. H), 7.05–7.18 (m, 2H, arom. H), 7.66–7.74 (m, 1H, arom. H), 8.60 (s, 1H, N*H*); ^13^C-NMR (101 MHz, DMSO-*d*_6_) δ [ppm] = 8.90, 32.96 (2C), 55.42 (*C*H_3_), 24.61 (*C*H_2_), 114.90 (2C), 118.31, 119.17 (2C), 123.21, 125.34, 125.50 (*C*H), 42.56, 129.43, 149.21, 150.00, 155.28 (*C*), 175.42 (*C=*O); C_19_H_23_NO_3_ (313.40): calcd. C 72.84, H 7.40, N 4.47, found C 72.72, H 7.45, N 4.52; EI-MS: *m*/*z* (%): 313.1 [M]^+^ (51), 190.1 [M − 123]^+^ (100); HPLC: 98.0% at 254 nm, 98.3% at 280 nm; t_R_ = 6.69 min, t_M_(DMSO) = 1.06 min (ACN/H_2_O = 60:40), λ_max_ [nm] = 228; HPLC-gradient: 98.1%, t_R_ = 13.43 min, t_M_(DMSO) = 1.28 min.

*N-[2-(4-Methoxyphenoxy)phenyl]-3-methylbutanamide* (**13**): Purification by column chromatography (petroleum ether/ethyl acetate 4:1) yielded a colorless oil (303 mg, 1.01 mmol, 67%); IR (NaCl): $\bar{\nu }$ [cm^−1^] = 3426 (m, N-H), 3317 (m, br, N-H), 1679 (s, C=O); ^1^H-NMR: (600 MHz, DMSO-*d*_6_): δ [ppm] = 0.87 (d, *J* = 6.7 Hz, 6H, C*H*_2_), 1.99 (hept, *J* = 6.8 Hz, 1H, C*H*), 2.21 (d, *J* = 7.2 Hz, 2H, C*H*_2_), 3.73 (s, 3H, OC*H*_3_), 6.72–6.83 (m, 1H, arom. H), 6.89–7.01 (m, 4H, arom. H), 7.01–7.10 (m, 2H, arom. H), 7.77–8.09 (m, 1H, arom. H), 9.35 (s, 1H, N*H*); ^13^C-NMR (151 MHz, DMSO-*d*_6_) δ [ppm] = 22.12 (2C), 55.32 (*C*H_3_), 44.94 (*C*H_2_) 25.60, 114.81 (2C), 117.42, 120.01 (2C), 122.71, 123.95, 124.65 (*C*H), 129.16, 148.63, 149.70, 155.35 (*C*), 170.87 (*C=*O); C_18_H_21_NO_3_ (299.37): calcd. C 72.22, H 7.07, N 4.68, found C 71.88, H 7.33, N 4.52; EI-MS: *m*/*z* (%): 299.1 [M]^+^ (48), 176.1 [M − 123]^+^ (100); HPLC: 98.5% at 254 nm, 98.4% at 280 nm; t_R_ = 4.37 min, t_M_(DMSO) = 1.06 min (ACN/H_2_O = 60:40), λ_max_ [nm] = 248; HPLC-gradient: 98.1%, t_R_ = 12.47 min, t_M_(DMSO) = 1.28 min.

*N-[2-(4-Methoxyphenoxy)phenyl]propanamide* (**14**): Crystallization from ethanol (70%) yielded colorless crystals (379 mg, 1.40 mmol, 93%); m.p.: 107–109 °C; IR (KBr): $\bar{\nu }$ [cm^−1^] = 3299 (m, br, N-H), 1651 (s, C=O); ^1^H-NMR: (400 MHz, DMSO-*d*_6_): δ [ppm] = 1.02 (t, *J* = 7.6 Hz, 3H, C*H*_3_), 2.35 (q, *J* = 7.5 Hz, 2H, C*H*_2_), 3.74 (s, 3H, OC*H*_3_), 6.70–6.79 (m, 1H, arom. H), 6.90–7.09 (m, 6H, arom. H), 7.87–8.10 (m, 1H, arom. H), 9.33 (s, 1H, N*H*); ^13^C-NMR (101 MHz, DMSO-*d*_6_) δ [ppm] = 9.68, 55.39 (*C*H_3_), 29.09 (*C*H_2_), 114.89 (2C), 117.23, 120.24 (2C), 122.70, 123.53, 124.45 (*C*H), 129.26, 148.49, 149.64, 155.48 (*C*), 172.28 (*C=*O); C_16_H_17_NO_3_ (271.32): calcd. C 70.83, H 6.32, N 5.16, found C 70.70, H 6.37, N 5.12; EI-MS: *m*/*z* (%): 271.1 [M]^+^ (54), 215.1 [M − 59]^+^ (100); HPLC: 98.6% at 254 nm, 97.9% at 280 nm; t_R_ = 4.89 min, t_M_(DMSO) = 1.06 min (ACN/H_2_O = 50:50), λ_max_ [nm] = 239; HPLC-gradient: 97.5%, t_R_ = 11.41 min, t_M_(DMSO) = 1.28 min.

*N-*[2-(4-Methoxyphenoxy)phenyl]*cyclopropanecarboxyamide* (**15**): Crystallization from ethanol (70%) yielded a colorless solid (360 mg, 1.27 mmol, 85%); m.p.: 138–139 °C; IR (KBr): $\bar{\nu }$ [cm^−1^] = 3312 (m, br, N-H), 1660 (s, C=O); ^1^H-NMR: (400 MHz, DMSO-*d*_6_): δ [ppm] = 0.65–0.87 (m, 4H, C*H*_2_), 1.95–2.14 (m, 1H, C*H*), 3.75 (s, 3H, OC*H*_3_), 6.64–6.81 (m, 1H, arom. H), 6.88–7.06 (m, 6H, arom. H), 7.88–8.05 (m, 1H, arom. H), 9.69 (s, 1H, N*H*); ^13^C-NMR (101 MHz, DMSO-*d*_6_) δ [ppm] = 55.39 (*C*H_3_), 7.25 (2C, *C*H_2_), 14.02, 114.93 (2C), 116.93, 120.47 (2C), 122.57, 123.38, 124.32 (*C*H), 129.21, 148.38, 149.54, 155.56 (*C*), 172.04 (*C=*O); C_17_H_17_NO_3_ (283.33): calcd. C 72.07, H 6.05, N 4.94, found C 71.86, H 6.20, N 4.93; EI-MS: *m*/*z* (%): 283.1 [M]^+^ (43), 215.1 [M − 68]^+^ (100); HPLC: 99.2% at 254 nm, 99.4% at 280 nm; t_R_ = 5.79 min, t_M_(DMSO) = 1.06 min (ACN/H_2_O = 50:50), λ_max_ [nm] = 247; HPLC-gradient: 98.6%, t_R_ = 11.73 min, t_M_(DMSO) = 1.28 min.

*N-[2-(4-Methoxyphenoxy)phenyl]cyclobutanecarboxyamide* (**16**): Crystallization from ethanol (70%) yielded a colorless solid (378 mg, 1.27 mmol, 85%); m.p.: 72–74 °C; IR (KBr): $\bar{\nu }$ [cm^−1^] = 3294 (m, br, N-H), 1665 (s, C=O); ^1^H-NMR: (400 MHz, CDCl_3_): δ [ppm] = 1.82–2.07 (m, 2H, C*H*_2_), 2.13–2.27 (m, 2H, C*H*_2_), 2.28–2.45 (m, 2H, C*H*_2_), 3.08–3.29 (m, 1H, C*H*), 3.81 (s, 3H, OC*H*_3_), 6.74 (dd, *J* = 8.1 Hz, 1.4 Hz, 1H, arom. H), 6.84–7.01 (m, 5H, arom. H), 7.06 (td, *J* = 7.8 Hz, 1.4 Hz, 1H, arom. H), 7.71 (s, 1H, N*H*), 8.47 (dd, *J* = 8.1 Hz, 1.6 Hz, 1H, arom. H); ^13^C-NMR (101 MHz, CDCl_3_) δ [ppm] = 55.67 (*C*H_3_) 18.02, 25.32 (2C) (*C*H_2_), 41.10, 114.99 (2C), 116.31, 120.29 (2C), 120.51, 123.35, 123.54 (*C*H) 129.29, 146.55, 149.48, 156.21 (*C*), 173.22 (*C=*O); C_17_H_17_NO_3_ (283.33): calcd. C 72.07, H 6.05, N 4.94, found C 71.86, H 6.20, N 4.93; EI-MS: *m*/*z* (%): 297.1 [M]^+^ (38) 215.1 [M − 82]^+^ (100); HPLC: 99.3% at 254 nm, 99.2% at 280 nm; t_R_ = 4.15 min, t_M_(DMSO) = 1.06 min (ACN/H_2_O = 60:40), λ_max_ [nm] = 248; HPLC-Gradient: 98.5%, t_R_ = 12.93 min, t_M_(DMSO) = 1.28 min.

*Methyl 4-{[2-(4-methoxyphenoxy)phenyl]amino}-4-oxobutanoate* (**17**): Crystallization from methanol yielded slightly yellow crystals (347 mg, 1.05 mmol, 70%); m.p.: 82–83 °C; IR (KBr): $\bar{\nu }$ [cm^−1^] = 3335 (m, br, N-H), 1721 (s, C=O, ester), 1682 (s, C=O, amide); ^1^H-NMR: (400 MHz, DMSO-*d*_6_): δ [ppm] = 2.55 (t, *J* = 6.8 Hz, 2H, C*H*_2_), 2.66 (t, *J* = 6.8 Hz, 2H, C*H*_2_), 3.58 (s, 3H, COOC*H*_3_), 3.75 (s, 3H, OC*H*_3_), 6.67–6.80 (m, 1H, arom. H), 6.86–7.12 (m, 6H, arom. H), 7.84–8.05 (m, 1H, arom. H), 9.52 (s, 1H, N*H*); ^13^C-NMR (101 MHz, DMSO-*d*_6_) δ [ppm] = 51.27, 55.39 (*C*H_3_), 28.61, 30.62 (*C*H_2_), 114.90 (2C), 117.00, 120.43 (2C), 122.60, 123.19, 124.41 (C*H*), 129.06, 148.39, 149.54, 155.54 (*C*), 170.19, 172.77 (*C=*O); C_18_H_19_NO_5_ (329.35): calcd. C 65.64, H 5.82, N 4.25, found C 65.52, H 5.91, N 4.12; EI-MS: *m*/*z* (%): 329.1 [M]^+^ (32), 297.1 [M − 32]^+^ (100); HPLC: 98.0% at 254 nm, 98.2% at 280 nm; t_R_ = 4.39 min, t_M_(DMSO) = 1.06 min (ACN/H_2_O = 50:50), λ_max_ [nm] = 245; HPLC-gradient: 97.4%, t_R_ = 11.20 min, t_M_(DMSO) = 1.28 min.

*N-(2-(4-Methoxyphenoxy)phenyl)-4-methoxybenzamide* (**18**): Crystallization from methanol yielded colorless needles (344 mg, 0.98 mmol, 66%); m.p.: 88–89 °C; IR (KBr): $\bar{\nu }$ [cm^−1^] = 3442 (m, N-H), 1674 (s, C=O); ^1^H-NMR: (400 MHz, DMSO-*d*_6_): δ [ppm] = 3.72 (s, 3H, OC*H*_3_), 3.82 (s, 3H, OC*H*_3*3*_), 6.80–6.88 (m, 1H, arom. H), 6.90–6.96 (m, 2H, arom. H), 6.96–7.04 (m, 4H, arom. H), 7.06–7.20 (m, 2H, arom. H), 7.76 (dd, *J* = 7.5 Hz, 2.1 Hz, 1H, arom. H), 7.84–7.93 (m, 2H, arom. H), 9.61 (s, 1H, N*H*); ^13^C-NMR (101 MHz, DMSO-*d*_6_) δ [ppm] = 55.37 (2C, *C*H_3_), 113.57 (2C), 114.88 (2C), 117.63, 120.08 (2C), 122.78, 125.89, 126.05, 129.45 (2C) (*C*H), 126.48, 128.93, 149.69, 150.56, 155.44, 161.87 (*C*), 164.68 (*C=*O); C_21_H_19_NO_4_ (349.39): calcd. C 72.19, H 5.48, N 4.01, found C 72.01, H 5.46, N 3.90; EI-MS: *m*/*z* (%): 349.1 [M]^+^ (20), 135.0 [M − 214]^+^ (100); HPLC: 99.4% at 254 nm, 99.4% at 280 nm; t_R_ = 4.71 min, t_M_(DMSO) = 1.06 min (ACN/H_2_O = 60:40), λ_max_ [nm] = 268; HPLC-gradient: 98.8%, t_R_ = 12.78 min, t_M_(DMSO) = 1.28 min.

*tert-Butyl 2-{[2-(4-chlorophenoxy)phenyl]amino}-2-oxoethylcarbamate* (**9**)

A stirred solution of Boc-glycine (1.50 mmol), PyBOP (1.60 mmol) and DIPEA (3.50 mmol) in dichloromethane (10 mL) was cooled to 0 °C with an ice bath. To this solution 2-(4-chlorophenoxy)aniline (1.50 mmol), dissolved in a minimum amount of dichloromethane, was added dropwise. The reaction mixture was allowed to warm to room temperature and was then stirred for 24 h. Thereafter, an aqueous work-up was performed similar to that of compounds **1**–**8**. The resulting oil was further purified by column chromatography over silica gel (dichloromethane/methanol 200:1) to give a slightly orange solid (358 mg, 0.95 mmol, 63%); m.p.: 50–51 °C; IR (KBr): $\bar{\nu }$ [cm^−1^] = 3409 (m, N-H), 3342 (s, N-H), 1691 (br, s, C=O); ^1^H-NMR: (600 MHz, DMSO-*d*_6_): δ [ppm] = 1.34 (s, 9H, C*H*_3_), 3.71 (d, *J* = 6.1 Hz, 2H, C*H*_2_), 6.87–7.06 (m, 3H, arom. H), 7.05–7.30 (m, 3H, arom. H), 7.35–7.53 (m, 2H, arom. H), 8.12 (d, *J* = 7.7 Hz, 1H, N*H*), 9.33 (s, 1H, N*H*); ^13^C-NMR (151 MHz, DMSO-*d*_6_) δ [ppm] = 28.00 (3C, *C*H_3_), 43.92 (*C*H_2_), 118.82, 119.90 (2C), 122.35, 124.15, 124.63, 129.70 (2C) (*C*H), 78.17, 127.17, 129.64, 146.09, 155.47, 155.86, 168.51 (*C*); EI-MS: *m*/*z* (%): 376.1 [M]^+^ (11), 219.1 [M − 157]^+^ (100); HREI-MS: calcd. for C_19_H_21_ClN_2_O_4_ 376.11844, found 376.11803; HPLC: 96.3% at 254 nm, 96.3% at 280 nm; t_R_ = 4.84 min, t_M_(DMSO) = 1.06 min (ACN/H_2_O = 60:40), λ_max_ [nm] = 230; HPLC-gradient: 95.8%, t_R_ = 12.80 min, t_M_(DMSO) = 1.28 min.

*tert-Butyl 2-{[2-(4-methoxyphenoxy)phenyl]amino}-2-oxoethylcarbamate* (**19**)

A stirred solution of Boc-glycine (2.00 mmol), PyBOP (2.10 mmol) and DIPEA (5.00 mmol) in dichloromethane (10 mL) was cooled to 0 °C with an ice bath. To this solution, 2-(4-methoxyphenoxy)aniline hydrochloride (2.00 mmol), dissolved in dichloromethane (5 mL) together with DIPEA (2.00 mmol), was added dropwise. The reaction mixture was allowed to warm to room temperature and was then stirred for 20 h. Subsequently, an aqueous work-up was performed similar to that of compounds **1**–**8**. The resulting oil was taken up in cold diethyl ether (10 mL) leading to precipitation of a beige solid, which was filtered off and discarded. The ether was evaporated under reduced pressure and the resulting residue was recrystallized from methanol/water (5:1) to give a slightly beige solid(495 mg, 1.33 mmol, 66%); m.p.: 92–93 °C; IR (KBr): $\bar{\nu }$ [cm^−1^] = 3393 (w, N-H), 3313 (s, br, N-H), 1696 (s, C=O, carbamate), 1666 (s, C=O, amide); ^1^H-NMR: (600 MHz, DMSO-*d*_6_): δ [ppm] = 1.35 (s, 9H, C(C*H*_3_)_3_), 3.73–3.75 (m, 5H, C*H*_2_, OC*H*_3_), 6.73 (dd, *J* = 8.0 Hz, 1.7 Hz, 1H, arom. H), 6.95–7.07 (m, 6H, arom. H), 7.24 (t, *J* = 5.2 Hz, 1H, arom. H), 8.13 (d, *J* = 7.0 Hz, 1H, N*H*), 9.30 (s, 1H, N*H*); ^13^C-NMR (151 MHz, DMSO-*d*_6_) δ [ppm] = 28.02 (3C), 55.33 (*C*H_3_), 44.06 (*C*H_2_), 114.92 (2C), 116.55, 120.52 (2C), 121.58, 122.67, 124.19 (*C*H), 78.22 128.63, 147.69, 149.16, 155.65, 155.90, 168.43 (*C*); C_20_H_24_N_2_O_5_ (372.42): calcd. C 64.50, H 6.50, N 7.52, found C 64.10, H 6.68, N 7.61; EI-MS: *m*/*z* (%): 372.2 [M]^+^ (18), 215.1 [M − 157]^+^ (100); HPLC: 96.5% at 254 nm, 95.5% at 280 nm; t_R_ = 3.41 min, t_M_(DMSO) = 1.06 min (ACN/H_2_O = 60:40), λ_max_ [nm] = 241; HPLC-gradient: 95.1%, t_R_ = 11.99 min, t_M_(DMSO) = 1.28 min.

#### General Procedure for the Synthesis of Compounds **10** and **20**

The reaction was performed under nitrogen atmosphere. The Boc-protected amine **9** (490 mg, 1.31 mmol) or **19** (250 mg, 0.67 mmol) was dissolved in dichloromethane (10 mL). The solution was cooled with an ice bath. To the well stirred solution 10 equivalents of trifluoroacetic acid (1.01 mL, 13.1 mmol or 0.516 mL, 6.70 mmol) were added dropwise. The reaction mixture was allowed to warm to room temperature and was stirred for 24h. Afterwards, the solvent was evaporated under reduced pressure. The resulting residue was taken up with propan-2-ol (4–5 mL). Then a mixture of hydrochloric acid (37%) and propan-2-ol (1:1) was added dropwise until a precipitate was formed. Thereafter, diethyl ether (20–30 mL) was added and the mixture was refluxed for 2 h. After cooling to room temperature the formed precipitate was filtered off and dried under reduced pressure at 60 °C.

*2-{[2-(4-Chlorophenoxy)phenyl]amino}-2-oxoethan-1-aminium chloride* (**10**): Crystallization from diethyl ether/propan-2-ol yielded a beige solid (220 mg, 0.70 mmol, 54%); m.p.: 255–257 °C (decomp.); IR (KBr): $\bar{\nu }$ [cm^−1^] = 3434 (m, br, N-H), 3124 (m, N-H), 1673 (s, C=O); ^1^H-NMR: (600 MHz, DMSO-*d*_6_): δ [ppm] = 3.78 (s, 2H, C*H*_2_), 6.95 (dd, *J* = 8.0 Hz, 1.6 Hz, 1H, arom. H), 7.01–7.05 (m, 2H, arom. H), 7.13–7.21 (m, 2H, arom. H), 7.42–7.48 (m, 2H, arom. H), 8.03 (dd, *J* = 8.0 Hz, 1.9 Hz, 1H, arom. H), 8.14 (s, 3H, N*H*_3_*^+^*), 10.12 (s, 1H, N*H*); ^13^C-NMR (151 MHz, DMSO-*d*_6_) δ [ppm] = 40.91 (*C*H_2_), 118.99, 120.12 (2C), 123.27, 124.12, 125.52, 129.72 (2C) (*C*H), 127.22, 128.90, 146.92, 155.45 (*C*), 165.48 (*C=*O); C_14_H_13_ClN_2_O_2_∙HCl (313.18): calcd. C 53.69, H 4.51, N 8.95, found C 53.89, H 4.51, N 8.57; EI-MS: *m*/*z* (%): 276.0 [M_free base_]^+.^ (36), 219.0 [M − 54]^+^ (100); HPLC: 99.9% 254 nm, 99.8% at 280 nm; t_R_ = 3.41 min, t_M_(DMSO) = 1.06 min (ACN/Buffer = 30:70), λ_max_ [nm] = 231.

*2-{[2-(4-Methoxyphenoxy)phenyl]amino}-2-oxoethan-1-aminium chloride* (**20**): Crystallization from diethyl ether/propan-2-ol yielded a beige solid (189 mg, 0.61 mmol, 91%); m.p.: 249–250 °C (decomp.); IR (KBr): $\bar{\nu }$ [cm^−1^] = 3434 (m, br, N-H), 3114 (m, N-H), 1680 (s, C=O); ^1^H-NMR: (600 MHz, DMSO-*d*_6_): δ [ppm] = 3.76 (s, 2H, OC*H*_3_), 3.84 (s, 2H, C*H*_2_), 6.72–6.77 (m, 1H, arom. H), 6.95–7.04 (m, 4H, arom. H), 7.05–7.13 (m, 2H, arom. H), 7.88–8.14 (m, 1H, arom. H), 8.26 (s, 3H, N*H*_3_*^+^*), 10.11 (s, 1H, N*H*); ^13^C-NMR (151 MHz, DMSO-*d*_6_) δ [ppm] = 55.35 (*C*H_3_), 40.85 (*C*H_2_), 114.95 (2C), 116.75, 120.73 (2C), 122.61, 122.82, 125.16 (*C*H), 127.88, 148.66, 149.11, 155.69 (*C*), 165.28 (*C=*O); C_15_H_16_N_2_O_3_∙HCl (308.76): calcd. C 58.35, H 5.55, N 9.07, found C 58.28, H 5.52, N 8.82; EI-MS: *m*/*z* (%): 272.1 [M_free base_]^+.^ (55), 200.1 [M − 72]^+^ (100); HPLC: 99.2% at 254 nm, 99.3% at 280 nm; t_R_ = 7.89 min, t_M_(DMSO) = 1.06 min (ACN/Buffer = 20:80), λ_max_ [nm] = 229.

#### 3.3. Calculations of Lipinski Properties

Calculations of the octanol/water partition coefficients (logP) were performed using chemicalize.org [15]. The calculator plugins are based on Viswanadhan’s fragmentation methods [22], the PHYSPROP^©^ database [23] and an additional data set [24]. All three approaches were weighted equally for the calculations.

#### 3.4. In Vitro Antimalarial Activity Assay

Erythrocytic stages of transgenic NF54-*luc P. falciparum* were used for the luciferase-based viability assay. These parasites constitutively express high levels of luciferase. The parasites were cultured as described earlier [19,25]. Firstly, the culture was dispensed in triplicate into white 96-well flat bottom plates (each well contains 250 µL) (NUNC, Roskilde, Denmark) with parasitemia of 0.5%–1%. Then the cultures were incubated in the presence of a 3 µM concentration of the test compounds for 48 h (37 °C, 90% N_2_, 5% CO_2_, and 5% O_2_). Subsequently, 100 µL RPMI1640 media was removed from each well and a 100 µL volume of the Bright-Glo^®^ substrate solution was added to each well. One of the cleavage products of the reaction is light, which was measured by a FLUOROSKAN FL luminometer (Thermo) machine, thereby detecting the amount of living parasites. The experiments were repeated with an incubation of 96 h. Untreated cultures were used as negative controls and to calculate the inhibition rate (0% inhibition of parasite growth). Blasticidin (Sigma-Aldrich, St. Louis, MO, USA), a drug used for *selection* of transfected parasites, was included as a positive control on each plate and gave >90% inhibition of parasite growth at 2 µg/mL.

## 4. Conclusions

In conclusion, the previously published *N*-[2-(4-chlorophenoxy)phenyl]-2,2-dimethylpropan­amide (TCMDC-137332, **1**) [7] and 19 derivatives were synthesized in satisfactory to excellent yields and were fully characterized regarding identity and purity. The predicted antimalarial activity of the herein examined hit structure of the TCAMS (**1**) was not confirmed in biological evaluation experiments. The growth of *Plasmodium falciparum* NF54 strains was not diminished in the expected nanomolar concentration by the 2-phenoxyanilide congeners. However, in a prolonged *in vitro* assay, in which cultures were incubated for 96 h, *N*-[2-(4-methoxyphenoxy)phenyl]cyclobutanecarboxyamide (**16**) was identified to be a moderate inhibitor of the proliferation of *Plasmodium falciparum* NF54 strains.

## Figures and Tables

**Table 1:** Calculated logP values, the number of H-bond acceptors (H_acc_) and donors (H_don_) and the molecular weight (MW) of selected compounds.

ID	Z	R	logP	H_acc_	H_don_	MW
1	Cl	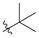	5.11	3	1	303.79
11	OCH_3_		4.35	4	1	299.37
4	Cl	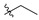	4.02	3	1	275.73
14	OCH_3_		3.25	4	1	271.32
5	Cl	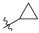	4.10	3	1	287.74
15	OCH_3_		3.33	4	1	283.33
7	Cl	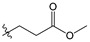	3.29	5	1	333.77
17	OCH_3_		2.52	6	1	329.35
10	Cl	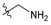	2.39	3	3	276.72
20	OCH_3_		1.63	4	3	272.30
